# Abstracts

**Published:** 1912-09

**Authors:** 


Salvarsan has given a negative result in a case of chronic leprosy. Gioseffi, *Gaz. degli Osp.*, Jan. 29, 1911. Experience of this sort is of special interest, since it may have occurred to many physicians to learn whether 606 has an antiseptic action in infectious processes of non-syphlitic origin. An amusing military case may be cited. The patient had obstinate amœbic dysentery. Becoming discouraged, he left the post hospital and consulted an up-to-date quack who diagnosed syphilis, injected 606 and cured the dysentery promptly.

Klemperer (*Deutsch. med. Woch.*, May 9th) gave salvarsan internally in an epidemic of scarlatina with a mortality of nearly 25 per cent. The mortality in the salvarsan series was about 8 per cent. In every case the injection was followed at once by a fall of temperature, a phenomenon never seen in any of the forms of sepsis, rheumatism, or tuberculosis.

